# Cytoskeletal Regulation Dominates Temperature-Sensitive Proteomic Changes of Hibernation in Forebrain of 13-Lined Ground Squirrels

**DOI:** 10.1371/journal.pone.0071627

**Published:** 2013-08-09

**Authors:** Allyson G. Hindle, Sandra L. Martin

**Affiliations:** Cell and Developmental Biology, University of Colorado School of Medicine, Aurora, Colorado, United States of America; Karlsruhe Institute of Technology, Germany

## Abstract

13-lined ground squirrels, *Ictidomys tridecemlineatus*, are obligate hibernators that transition annually between summer homeothermy and winter heterothermy – wherein they exploit episodic torpor bouts. Despite cerebral ischemia during torpor and rapid reperfusion during arousal, hibernator brains resist damage and the animals emerge neurologically intact each spring. We hypothesized that protein changes in the brain underlie winter neuroprotection. To identify candidate proteins, we applied a sensitive 2D gel electrophoresis method to quantify protein differences among forebrain extracts prepared from ground squirrels in two summer, four winter and fall transition states. Proteins that differed among groups were identified using LC-MS/MS. Only 84 protein spots varied significantly among the defined states of hibernation. Protein changes in the forebrain proteome fell largely into two reciprocal patterns with a strong body temperature dependence. The importance of body temperature was tested in animals from the fall; these fall animals use torpor sporadically with body temperatures mirroring ambient temperatures between 4 and 21°C as they navigate the transition between summer homeothermy and winter heterothermy. Unlike cold-torpid fall ground squirrels, warm-torpid individuals strongly resembled the homeotherms, indicating that the changes observed in torpid hibernators are defined by body temperature, not torpor per se. Metabolic enzymes were largely unchanged despite varied metabolic activity across annual and torpor-arousal cycles. Instead, the majority of the observed changes were cytoskeletal proteins and their regulators. While cytoskeletal structural proteins tended to differ seasonally, i.e., between summer homeothermy and winter heterothermy, their regulatory proteins were more strongly affected by body temperature. Changes in the abundance of various isoforms of the microtubule assembly and disassembly regulatory proteins dihydropyrimidinase-related protein and stathmin suggested mechanisms for rapid cytoskeletal reorganization on return to euthermy during torpor-arousal cycles.

## Introduction

Hibernation includes some of the most dramatic physiological changes tolerated by mammals. In ground squirrels, this natural feature of life history involves transition from a summer homeothermic to a winter heterothermic state. In winter, animals are capable of 1–2 week torpor bouts that are rapidly and repeatedly reversed to inter-bout arousal (IBA) [1]. Body temperature (T_b_) and vital signs drop precipitously during entrance into torpor, a period also associated with reduced metabolic rate and global ischemia [2], [3], [4]; hypoxemia is thought to be avoided during torpor as metabolic rate declines in concert with perfusion levels during entry into and maintenance of torpor to match oxygen delivery with demand. Small-bodied hibernators arouse from torpor at near-zero T_b_ to euthermic T_b_ via endogenous heat production mechanisms [1]. Rewarming episodes, in contrast to periods of torpor, represent periods of intense metabolic activity, high oxygen demand and restoration of perfusion to ischemic regions. The system is therefore challenged during rewarming when metabolic rates rise in advance of perfusion, leading to tissue hypoxemia and ischemia-reperfusion events [3], [5]. Yet, these hibernators reversibly orchestrate torpor-arousal cycles throughout winter with no observable long-term tissue damage [5].

The brain is a central regulator of hibernation [6]. Yet, brain tissue experiences the temperature, oxygenation and metabolic rate declines associated with a reduced homeostatic set point during torpor [6], [7], [8], making damage-free recovery from the torpid state paramount. Indeed, evidence for neuroprotection in ground squirrel brain during hibernation, combined with rapid transitions between torpor and euthermy, suggest an innate adaptation to protect and maintain function in the brain during extreme physiological challenge [5]. Such defenses suggest that hibernator brains provide a unique model system for uncovering novel strategies of mitigating damage due to neurodegeneration and cerebral ischemia in humans [5], [9].

Among the components of neuroprotection in the hibernator are: 1) depressed metabolic rate reduces oxygen demand to match the ∼90% reduction of cerebral perfusion during torpor [7]; 2) a mechanism for cytoskeletal stabilization, including synapses, during torpor allows rapid cytoskeletal reorganization on return to euthermy [5]; and 3) cellular homeostasis is maintained at low T_b_ [10]. Reduced T_b_ is associated with neural retraction across multiple brain regions in mammals: cell bodies, dendritic spines and synaptic profiles shrink [11], accompanied by general cytoskeletal breakdown as microtubules disassemble [12]. Yet, the hibernator’s brain is capable of rapid dendritic regrowth and synaptic rebuilding during each interbout arousal [13], [14]. Entry into torpor is associated with 50–60% loss of synapses in golden-mantled ground squirrel (*Callospermophilus lateralis*), the result of decreased co-localization of pre- and post-synaptic protein markers, although their abundance does not change [15]. The disassembly of synapses occurring with each torpor bout likely complements channel arrest to reduce the potential for Ca^2+^ buildup at cold T_b_ and detrimental excitotoxicity [5]. Synaptic protein disassociation at low T_b_ may also create a pool from which to draw building blocks for synaptic regeneration upon arousal [15]. Although the phenomenon of dendritic retraction in hibernators is well described, the molecular components that enable rapid and reversible cytoskeletal and synaptic reorganization with each torpor-arousal cycle are unknown.

Here, we set out to define proteomic changes in the forebrain of 13-lined ground squirrels that occur in concert with physiological adjustments. The rationale behind this approach was that protein changes associated with neuroprotective elements supporting torpor-arousal cycles could be revealed by defining differences to alternative states. Brain proteomic variations in multiple stages of hibernation have not yet been documented. Six physiologically defined states encompassing summer, spring and the torpor-arousal cycle of winter were analyzed in addition to a group of fall animals transitioning from summer homeothermy to winter heterothermy. This experiment was intended to capture the broad-scale defense strategies employed across a variety of cell types to contend with T_b_ and metabolic rate declines during torpor. Two significant aspects of these data shed light on proteomic regulation in the forebrain of 13-lined ground squirrels. Body temperature segregates the abundance of many proteins, indicating distinct strategies for maintaining homeostasis in torpor compared to interbout arousals and summer homeothermy. There is also a dominant cytoskeletal signature among the proteins that change across the hibernator’s year, indicating its importance in neuroprotection.

## Methods

### Tissue Collection

**Figure 1:**
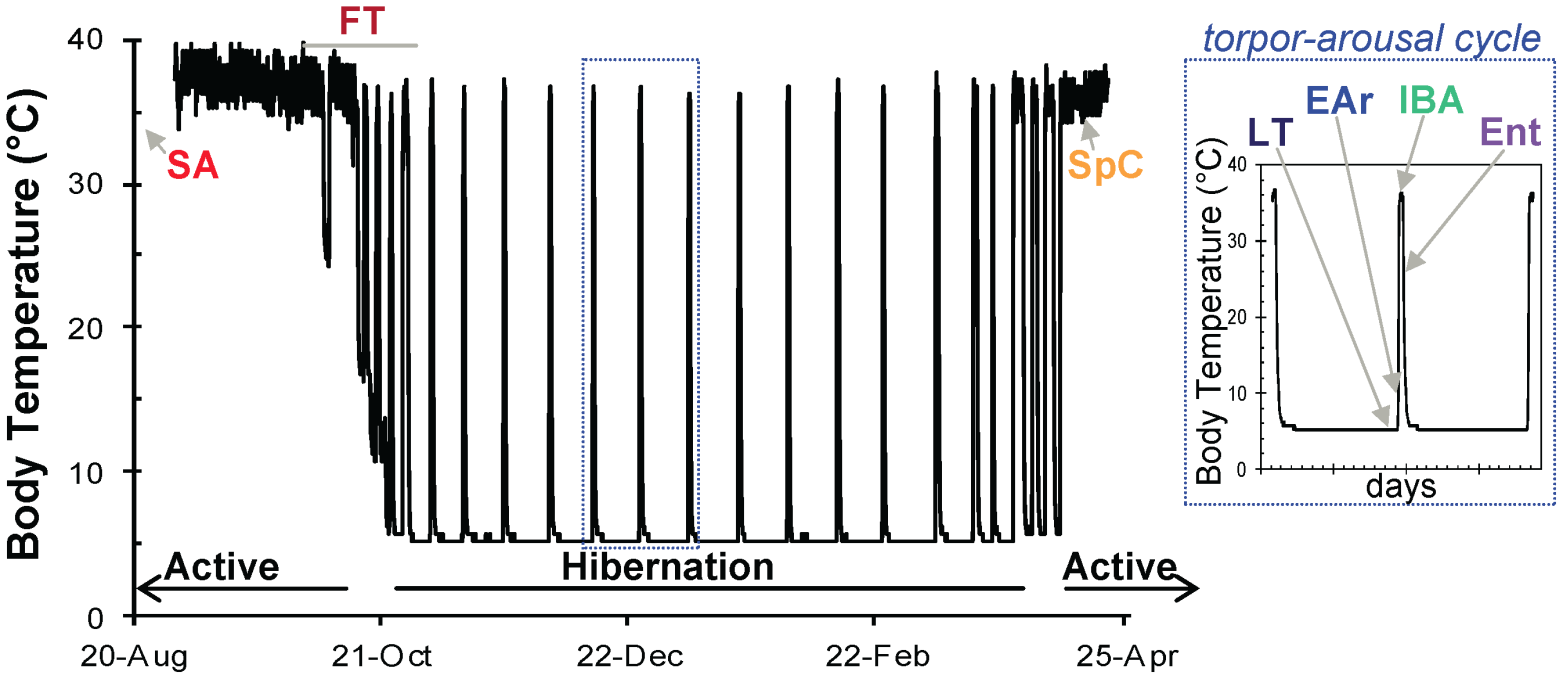
Sampled states of the hibernator’s year were defined by body temperature. Samples were collected from 7 states. Summer (SA, n = 6) and spring (SpC, n = 5) ground squirrels represented the homeothermic portions of the annual cycle. Winter states representing the heterothermic period (blue box, expanded for clarity) were collected throughout the winter hibernation season and were defined as follows by body temperature telemetry: entering torpor (Ent, n = 7) at 27°C>T_b_ >23°C; late torpor (LT, n = 7) at 80–95% previous bout duration; early arousing (EAr, n = 6) at 7°C<T_b_ <12.8°C; and 3–4 hours after T_b_ stabilized in inter-bout arousal (IBA, n = 5) period. Samples were also collected during the highly variable Fall transition period (FT, n = 12) [16]. Colors representing each state are used throughout.

Thirteen-lined ground squirrels purchased in July from the University of Wisconsin captive breeding program were housed at the University of Colorado individually at 18–21°C (14∶10 L:D) with dry cat food, sunflower seeds and water provided *ad libitum*. Figure 1 describes sampled states. Summer active (SA) ground squirrels were euthanized Aug. 8. The remaining ground squirrels were maintained in standard housing until Oct. 5 when they were transferred to the hibernaculum (4°C, 0∶24 L:D). To identify winter sampling states (Fig. 1) during hibernation, animals were each implanted with an intraperitoneal T_b_ recorder (iButton, Embedded Data Systems) and a telemeter (MiniMitter). Animals (IBA, Ent, LT, EAr) were collected during the specified T_b_ points or ranges (Fig. 1) within regular torpor-arousal cycles between 28-Dec and 26-Mar. Surgeries were performed under isoflurane anesthesia with post-operative analgesia with all efforts made to minimize suffering. Animals were transferred into the cold room only after a minimum 2-week post-surgical recovery. Food and water were removed once ground squirrels exhibited regular torpor in the cold room and returned to Spring animals (SpC) at least 10d before tissue collection. Samples were also collected from Fall Transition (FT) individuals between 21-Sept and 12-Oct to capture the physiological range of fall (FT animals were a mixture of individuals housed in standard laboratory housing or in the hibernaculum as or after the ambient temperature was lowered to 4°C and being torpid or not torpid at time of tissue collection [16]. Torpor in Fall Transition ground squirrels was evaluated from implanted T_b_ biologgers (iButton). Biometrics, housing and T_b_ data are presented in Table S1 for Fall individuals. This study was conducted according to the recommendations in the Guide for the Care and Use of Laboratory Animals of the National Institutes of Health. All animal care and procedures were approved by the University of Colorado School of Medicine Institutional Animal Care and Use Committee (protocol #44309).

For tissue collection, ground squirrels were anesthetized with isoflurane, then exsanguinated via cardiac blood draw and perfused with 6 volumes of ice-cold saline. After decapitation, brains were removed and placed on ice, then brainstem with cerebellum, olfactory bulbs and hypothalamus were dissected free. The remaining brain tissue, consisting of forebrain minus the hypothalamus was immediately frozen in N_2_(*l*), then stored at −80°C until processing. This sample was hemisected, and the right hemisphere was pulverized in N_2_(*l*) prior to bead-homogenization on ice (Bullet Blender, Next Advance) and centrifugation to retain the soluble, non-nuclear fraction as previously described [17]. Protein concentration of the homogenate was determined using a BCA assay (Pierce).

### 2D Gel Analysis and Protein Identification

The procedure for processing and labeling sample homogenates of equal protein content with Cy2, 3 or 5 fluors (GE Healthcare) and their separation on 2D gels were as described [18]. 24 gels, each containing 2 experimental samples (Cy3- and 5-labeled) and a pooled reference sample (Cy2-labeled) were imaged and imported along with Sypro Ruby stained pick gel images into DeCyder software (V7.0, GE Healthcare) for analysis [18]. Protein spots in all Cy2 reference images were matched automatically after manually landmarking ∼400 spots. Each Cy3 and Cy5 spot from the 48 remaining gel images were matched and normalized for spot intensity corresponding to Cy2.

We used one-way ANOVA with false-discovery rate correction [19] to identify protein spots whose abundance differed significantly by hibernation state. Only spots present in ≥3 experimental samples from each state were included. Spots with ANOVA q<0.05 after FDR correction were examined on all gels and manually matched in additional samples where possible. 77% of protein spots ultimately identified from this assessment had abundance data for all sampled animals and a given sampled state was left with only 3 data points (3 of 6 possible) only once. Hibernation states were also classified with the machine-learning clustering tool, Random Forests [20], [21]. The minimum required protein spots of known identity present on all gels that produced the lowest out-of-bag clustering error between the states were identified by Random Forests variable section algorithm (drop = 0.2, n = 100,000 iterations) [22]. All analyses were conducted on two datasets – the six base hibernation states with and without the Fall Transition – and were performed in R (v2.15.0, http://www.r-project.org) [23].

We used LC-MS/MS to attempt identification of protein spots that were significant by ANOVA after FDR or by Random Forests. Gel plugs (diameter = 1.4****mm, depth = 1****mm) containing spots of interest and that were reproducible on the pick gels were picked and digested with an automated system as previously described [17]. We collected spectral data from tandem MS on the four highest peaks with 30s dynamic exclusion as previously described [17]. Tryptic fragments were analyzed with Spectrum Mill MS Proteomics Workbench (revision B.04.00, Agilent Technologies) against a protein database containing predicted sequences from the published 13-lined ground squirrel genome (Ensembl build 67, 20 000 entries, 1 missed residue allowed). Spots not identifiable using this database were subsequently searched against the National Center for Biotechnology Information mammal sequences from April 2012 and previously published actual sequences from arctic ground squirrels, *Urocitellus parryii* [24], permitting 2 missed residues. Including the 13-lined ground squirrel genomic information, this database contained 1,071,259 entries. Settings such as precursor and fragment ion mass tolerances, allowed missed cleavages, and amino acid modifications followed Hindle et al. [17].

We retained protein identifications (IDs) with at least two supporting peptides and a score ≥30. Protein spots which resolved into a unique protein ID with ≥3 supporting peptides and a score ≥30 were considered identified from a single pick gel. Spots that did not initially resolve into a unique ID were analyzed from 1–3 additional gels. The resulting spectral data were merged with an in-house program, ExtracTags (v4.1). The top hit from multiple IDs was retained only when it presented a ≥4-fold higher score, peptide recovery or spectral intensity than any other hit. Single best IDs for each analysis are presented in Tables S2&S3 and the complete file output including unresolved weak and multiple IDs is given in Table S4.

### Additional Analyses & Data Visualization

Significant pairwise differences between individual hibernation states were determined by Tukeys HSD posthoc comparisons (Tables S5&S6). Data were visualized by heatmap and Random Forests MDS plots (n = 50,000 trees) based on all significant, identified protein spots and those determined to produce the lowest-error clusters, respectively. Protein spot clusters generated by heat map were submitted to the DAVID bioinformatics database (V6.7) to identify significantly enriched gene pathways [25], using settings as described [17]. The proximity of the Fall Transition proteome to the physiologically defined states was predicted with spot data from the six defined states as a training set in Random Forests [21] and tested using unsupervised classification.

We examined phosphoprotein staining as evidence of posttranslational protein modification (PTM) in forebrain samples from IBA and EAr animals (n = 3 per state). 150 µg of unlabeled protein from each sample were separated on 2D gels (DiGE protocol), fixed overnight (50% MeOH, 10% acetic acid), then stained for phospho-proteins (ProQ Diamond, Molecular Probes followed by total protein staining (SYPRO Ruby) as described [26]. Western blots confirmed total protein content in proteins of interest. 20 µg protein of IBA and LT ground squirrels (n = 3 per state) were separated with SDS-PAGE then transferred to PVDF membranes (Immobilon-FL, Millipore). Consistent protein loading and transfer were assessed with total protein stain (MemCode™ #24585, Pierce). After blocking (Odyssey blocking buffer, Li-Cor) blots were incubated overnight at 4°C with anti-DYPSL2 (1∶1000) or anti-STMN1 rabbit pAbs (Cell Signaling), then for 1.5 h with a secondary antibody for anti-rabbit IgG (1∶15,000 IRDye 800CW-conjugated, Li-Cor). Examination of serine phosphorylation was attempted by multiplexing immunoblots with mouse anti-pSer mAb (1∶500, 3C171 Abcam) with secondary detection via anti-mouse IgG (1∶15,000 IRDye 680RW-conjugated, Li-Cor). Blots were imaged with Odyssey near-infrared fluorescence detection (Li-Cor) and total protein densities were calculated in ImageJ (http://imagej.nih.gov/ij, National Institutes of Health, USA) and analyzed with ANOVA. 2D western blots following electrophoresis methods for phosphoprotein staining were also employed.

## Results

### Forebrain Proteomic Changes are Limited and Associated with Body Temperature

In six physiologically defined hibernation states of 13-lined ground squirrels (Fig. 1) we detected 84 protein spots (from 3471 total spots analyzed with DiGE) that differed significantly among groups. 56 of these were successfully identified by LC-MS/MS, which represented 34 unique proteins due to multiple isoforms (Table S2). When the Fall Transition animals were included in the ANOVA, 88 protein spots significantly differed (54 identified; Table S3). These two ANOVAs combined to suggest 98 protein spots of interest.

**Figure 2:**
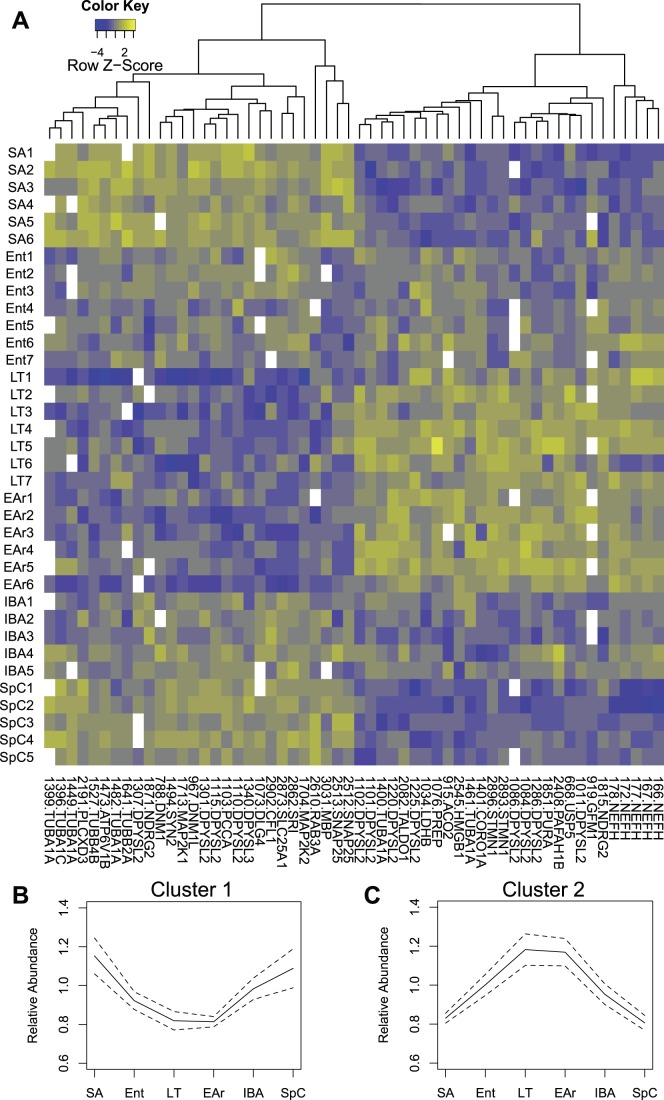
Forebrain proteome heat map. The forebrain proteome of cold-T_b_ ground squirrels demonstrated a reciprocal abundance pattern to euthermic animals. (A) Heat map y-axis: Individual ground squirrels grouped by state. Heat map x-axis (bottom): Protein spots (Spot Number.GeneID) that differed significantly by hibernation state (ANOVA q) and identified by LC-MS/MS were hierarchically clustered (X-axis (top): dendrogram). Blank squares in the heat map represent samples in which a given protein spot was not recovered. (B & C) Means with 95% confidence intervals are plotted for the two reciprocal clusters.

Hierarchical clustering of spot intensities and visualization by heatmap revealed two dominant reciprocal patterns of protein abundance (Fig. 2A). In cluster 1, spot abundance was highest in homeotherms (SA & SpC, Fig. 2B) and lowest in the cold-T_b_ states, LT and EAr. Warm- (IBA) or declining-T_b_ states (Ent) during winter resembled the homeothermic proteome or an intermediate abundance pattern, respectively. DAVID analysis of protein IDs belonging to cluster 2 (Fig. 2C) contained enrichments of proteins with roles in neural growth and differentiation and synaptic transmission (Table 1). Cluster 1 also revealed enrichment of the neurogenesis/differentiation pathway. Significantly, both clusters indicated differential regulation of the cytoskeleton (Table 1). The considerable protein overlap in the two clusters implies the importance of specific isoforms in regulating neural and cytoskeletal architecture during torpor-arousal cycles.

**Table 1:** Significant pathway enrichments in squirrel forebrain for two primary hierarchical clusters.

Annotation	Definition	Enrichment Score	Fold Enrichment	Genes
*Cluster 1*				
1	GTPase activity	3.0	18.5	RAB3A, DNM1L, TUBB2A, TUBA1A, DNM1, TUBA1C
2	Growth cone	2.2	35.5	DPYSL3, DPYSL2, SNAP25
3	Neuron differentiation	1.7	7.3	RAB3A, MAP2K1, TUBB2A, SNAP25
4	Endocytosis	1.7	10.9	RAB3A, DLG4, DNM1
5	Synaptic transmission	1.6	13.4	RAB3A, SYN2, DLG4, SNAP25, MBP
6	Neurotransmitter secretion	1.6	70.2	RAB3A, SYN2, SNAP25
7	Cytoskeleton Regulation	1.3	5.5	MAP2K1, MAP2K2, CFL1
*Cluster 2*				
1	Neurogenesis/differentiation	1.7	26.3	NDRG2, STMN1, DPYSL2
2	Cytoskeleton	1.6	4.0	CORO1A, NEFH, NDRG2, STMN1, DPYSL2, TUBA1A

Note. Hierarchical Cluster 1, with seven significantly enriched functional annotation clusters, contains proteins decreased in cold T_b_, whereas hierarchical Cluster 2, with two significant functional annotation clusters, contains proteins increased at cold T_b_, and decreased during euthermy (see Fig. 2). Enrichment score gives the mean *p*-value among members of each annotation cluster (negative log). Enrichment data were calculated by DAVID Bioinformatics Resources v6.7 [25].

**Figure 3:**
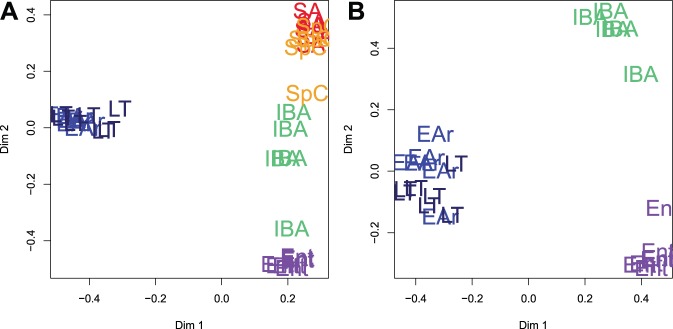
Sampled state separation by Random Forests. Data were clustered by Random Forests (50,000 trees) after variable selection to determine the minimum input protein spots required for the lowest classification error among groups. This was accomplished with (A) 22 protein spots for the base states and (B) 11 for winter states only (see Fig. 5).

**Figure 4:**
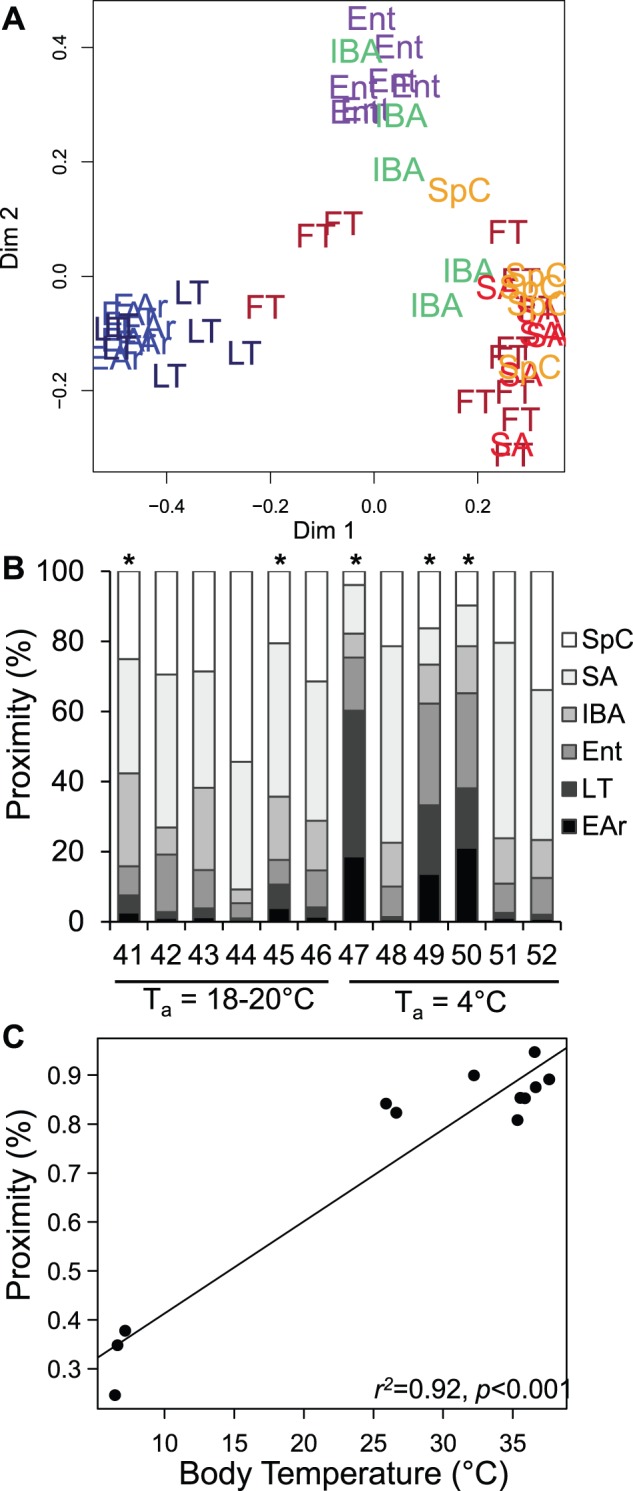
The fall proteome tracks body temperature. Fall transition individuals were held in warm (T_a_ = 18–20°C, #41–46) or cold rooms (T_a_ = 4°C, #47–52). (A) Unsupervised classification of all states using the most important predictors determined in Fig. 3A maintains the segregation of states by T_b_. The mixed physiology of Fall animals can test the effect of T_b_. (B) Unsupervised classification quantified proximity of FT individuals based on the defined groups as training data. In all but three individuals, more than 50% of the FT proteome was similar to summer or spring euthermic ground squirrels (x-axis: animal ID). The three exceptions were cold torpid (* denotes torpid), and instead had proteomes more proximate to LT/EAr. The absence of a similar pattern in the two warm-housed but torpid individuals (T_b_ = 18–21°C), supports the hypothesis that the LT/EAr proteome is defined by T_b_, and not torpor per se. (C) Proximity to the euthermic groups (SA+SpC+IBA) correlates significantly with T_b_.

Random Forests clustering segregates the hibernation states by T_b_ (Fig. 3). The two cold-T_b_ groups, LT and EAr, are not separated under any conditions, even when the winter states are examined alone (Fig. 3B). Indeed, only three protein spots significantly differ between these two groups (Tukey posthoc, Table S5). The segregation among sampled states persisted when Random Forests was run in unsupervised mode (Fig. 4A). The mixed-physiology FT animals were used to test whether separation among hibernation states resulted from changes in T_b_ or torpor status. We performed two assessments of Fall Transition (FT) individuals with unsupervised classification. Initially, 2D scaling plots were used to visualize the classification of FT individuals, which lie predominantly with the homeothermic states (Fig. 4A). Subsequently, unsupervised clustering of FT quantified proximities of the mixed-physiology animals to each defined state (Fig. 4B). All but three FT individuals (which were cold-torpid) had a forebrain proteome most proximate to the homeotherms (Figs. 4A&B). Two additional individuals using torpor in standard laboratory housing shared the high proximities to homeothermic states observed in non-torpid animals (Fig. 4B). A significant positive correlation between T_b_ at time of tissue collection and proximities to SA, or to the euthermic states overall (SA+SpC+IBA; Fig. 4C) suggests that T_b_ rather than torpor per se, is related to the brain protein patterns observed in LT and EAr.

### Cytoskeletal Proteins Dominate the Dataset

**Figure 5:**
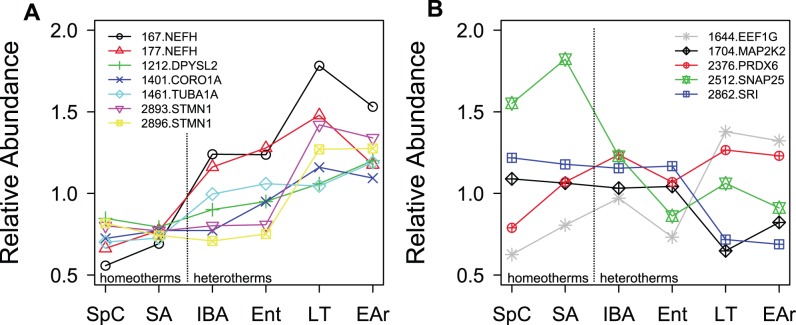
Abundance of protein spots important for separating clusters. The relative abundance of (A) cytoskeletal elements and binding proteins and (B) non-cytoskeletal proteins highlighted by Random Forests as important inputs for hibernation state clustering (Fig. 3). Mean values in each base state are plotted for spots that also had ≥1.5X maximum fold change. A broken vertical line divides the sequential homeothermic from heterothermic states.

Random Forests clustering also revealed protein spots of interest. A combined 23 protein spots were key in the lowest-error clustering for comparisons among hibernation states (Figs. 3A&B). 18 (78%) of these spots overlapped between this analysis and the ANOVA. Approximately half of the spots important in separating the states by Random Forests were cytoskeletal components or binding proteins (represented by GENE SYMBOL^master gel spot#^ below). Focusing on those with ≥1.5X maximum fold change (Fig. 5), the selected cytoskeletal proteins (Fig. 5A) generally increase from summer to winter. Two building blocks of nerve cell architecture, neurofilament heavy-chain (NEFH^167,177^) and α-tubulin isoform 1A (TUBA1A^1461^) displayed a general winter elevation compared to summer. The remainder, DYPSL2^1212^, CORO1A^1401^, STMN1^2983,2986^ (Fig. 5A), which are actin and tubulin binding proteins, show a strong increase in just LT and EAr. This pattern indicates regulation aimed at specifically managing low temperature and not the heterothermic winter state in general. Eukaryotic translation elongation factor 1 γ-subunit (EEF1G^1644^) shares this pattern of cold-T_b_ elevation (Fig. 5B). The inverse pattern (low in only LT/EAr) is born by both MAP kinase kinase 2 (MAP2K2^1704^) and sorcin (SRI^2862^). The abundance of synaptosomal-associated protein 25 (SNAP25^2512^), involved in Ca^2+^ synaptic responsiveness, peaks in SA and displays no significant pairwise differences among the winter states (Tables S5&S6). Peroxiredoxin 6 (PRDX6^2376^) is least abundant in spring (Fig. 5B), and also does not significantly differ among the winter groups (Tables S5&S6). A limited complement of metabolic enzymes were included in these defining proteins for the Random Forests clustering; transaldolase, (TALDO1^2084^) was elevated in LT and EAr, exhibiting the highest maximum fold change of four metabolic proteins, being 1.35-fold higher in EAr compared to SA (Table S7). Normalized abundance data for all identified spots are presented in Table S8.

### Posttranslational Modifications (PTMs) Support Abundance Shifts During Heterothermy and Torpor

The nature of intra-winter protein abundance cycles may suggest mechanisms of regulation in heterothermy. Increased gene expression by protein synthesis in the homeothermic season, or during euthermy between torpor bouts is the likely explanation for spot abundance increases during warm-T_b_ states such as SA, SpC, IBA and Ent. However, changes in subcellular sequestration or PTM are additional explanations for an increased relative protein spot abundance. A modification such as phosphorylation or acetylation creates isoelectric point shifts for protein isoforms that would result in a train of spots on 2D gels. If steady-state levels of a protein remain constant, we expect that PTMs conferring a change in protein activity should manifest as spots bearing the same ID but having reciprocal abundance patterns within a train. This dataset contains several examples of same-identity spots appearing in trains and bearing reciprocal patterns.

**Figure 6:**
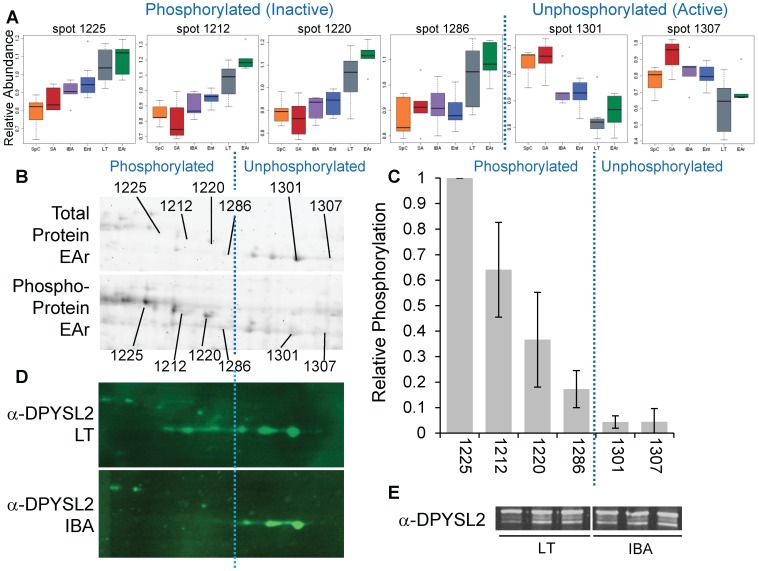
Posttranslational modification of a microtubule regulating protein. (A) Boxplots of the relative abundance of six DPYSL2 isoforms reveal two reciprocal abundance patterns; animal groups are as in Figure 1. (B) Representative images from an EAr 2D gel stained for total protein (top panel) or phosphoprotein (bottom panel); note the transition between the two patterns in panel A occurs at an isoelectric point consistent with a shift between the phosphorylated and unphosphorylated form of the protein. (C) Relative degree of isoform phosphorylation quantified from EAr and IBA phosphoprotein-stained gels increases as spots migrate further left on 2D gels (i.e., become more acidic), and supports categorizing the two abundance patterns by phosphorylation state. (D) Representative 2D western blots from LT and IBA show that the unphosphorylated forms are most abundant in euthermic states such as IBA, while phosphorylated forms are largely absent. (E) A 1D western blot (α-DPYSL2) shows no significant change in total DPYSL2 content among forebrain of LT, IBA or SA 13-lined ground squirrels (SA data not shown). In panels A–D the dotted blue line separates the phosphorylated and unphosphorylated forms of DPYSL2; in panels D–E, DPYSL2 antibody labeling is denoted as α-DPYSL2.

α-tubulin isoform 1A (TUBA1A) may appear winter-depleted (spots #1399, #1449, Table S7) or winter-elevated (spots #1400 and #1461, Fig. 5A, Table S7). Given that transcription and protein synthesis effectively cease at the cold-T_b_s of torpor [27], [28], spot abundance increases in the cold-T_b_ states of LT and EAr, a major pattern observed in this dataset (Fig. 2), are more likely to derive from changes in PTMs or perhaps subcellular sequestration. Dihydropyrimidinase-related protein 2 (DPYSL2) provides an example of an altered PTM. The six DPYSL2 isoforms present two inverse abundance patterns (Fig. 6A) despite no significant change in the total abundance of this protein (p = 0.72, Fig. 6E). The transition between these reciprocal patterns occurs at an isoelectric point consistent with an observed shift in phosphostaining (Fig. 6A–D), suggesting that the left-most spots are increasingly phosphorylated forms of DPYSL2 and that isoforms with this PTM accumulate at cold T_b_.

## Discussion

The brain of the hibernating ground squirrel endures repeated temperature, perfusion and metabolic shifts throughout the torpor-arousal cycles of winter [6], [7], [8]. In spite of these homeostatic challenges, hibernator brains regain functionality after each torpor bout and remain largely damage free in the face of reperfusion and rewarming [5].

Although the brain is known to play a regulatory role in hibernation, our understanding of underlying molecular mechanisms that support winter heterothermy is very limited. Here, we investigated the forebrain proteome of hibernating 13-lined ground squirrels in an effort to uncover the broad-scale defense and neuroprotection strategies associated with rapid morphological and functional recovery from regular torpor bouts throughout winter. We were able to identify specific proteins that are changed across the hibernator’s year. Surprisingly, the bulk of these changes respond to temperature oscillations, revealing two distinct protein complements underlying warm- versus cold-T_b_ states.

### Cell Function during Hibernation

The forebrain proteome in hibernating ground squirrels appears in several ways to be an exception among tissues studied to date. The magnitude and diversity of proteins recovered on 2D gels was the highest yet (3417 protein spots on the master gel), however, the degree of proteomic response across the hibernation cycle was minimal (only 84 protein spots or ∼2.5% differed significantly among the six physiologically defined states). Also, in contrast to previous proteomic studies of skeletal muscle, heart and kidney following the methods used here [17], [29], [30], there is a notable paucity of metabolic enzymes contained in the proportion of the brain proteome that varies with hibernation. Only four metabolic enzymes differed significantly among the hibernation states and were also identified by Random Forests as an important input for classifying the groups. Of these four, transaldolase had the largest fold change (1.35X, Table S7). This enzyme of the pentose phosphate pathway aids in connecting the non-oxidative portion of this energy-producing pathway with glycolysis. The pentose phosphate pathway provides cells with metabolites essential for nucleic acid synthesis such as ribose-5-phosphate and reducing cofactors such as NADPH [31]. Transaldolase abundance peaks early in the rewarming period, a time consistent with a need for elevated NADPH production to maintain the reduced glutathione pools that protect against oxidative damage [32].

The absence of sweeping changes in metabolic enzymes across the hibernation cycle as seen previously in tissues including liver [33], heart [29], skeletal muscle [17] and even brainstem [18] leads to the supposition that passive temperature-regulation of enzyme activity is important in the forebrain. In other words, cerebral metabolism is not actively suppressed in advance of torpor, but rather enzymatic processes are slowed via the Q_10_ effect as T_b_ declines [34]. Our results do not support the expected seasonal change in metabolic strategy in this tissue [3], perhaps highlighting the importance of circulating substrate delivery to brain, rather than tissue-level production [35]. This lack of evidence for substrate switching to accompany the winter fast is in contrast to seasonal mRNA expression in cerebral cortex consistent with ketone use and glucose conservation [36]. This difference could reflect post-transcriptional gene regulation or the difference in tissue complexity between the two studies.

Fall Transition individuals shared the majority of their forebrain proteome with summer and spring (Fig. 4). Prior experience with torpor, and in fact the collection of tissues during torpor bouts in standard laboratory housing when T_b_ was ∼22°C did not affect this similarity to the homeotherms. Rather, T_b_ or a physiological correlate seems to be a more significant seasonal mechanism of cell regulation in forebrain as opposed to a coordinated fall reprogramming to generate a proteome prepared for winter heterothermy, with subsequently minor changes observed across torpor-arousal cycles. This is evidenced by the proteomic similarity of fall cold- but not warm-torpid animals to those in winter torpor (LT) or rewarming (EAr) (Fig. 4). Overall, a shift between cold and warm T_b_ dominated the proteomic changes observed in 13-lined ground squirrel forebrain (Figs. 2&4). Again, this response is markedly different from multi-state proteomic examinations of other tissues (skeletal muscle, kidney, heart) conducted in our lab, in which a summer-winter shift comprised the bulk of protein changes [17], [29], [30].

While control of metabolic enzyme activity during torpor may derive largely from passive effects of low T_b_, the strong signature of temperature oscillation in other proteins suggests that distinct homeostatic regulatory events do occur. Sorcin (soluble resistance-related calcium binding protein) may be one such example. The abundance of sorcin (SRI^2962^) is decreased during cold-T_b_ states (Fig. 5B). Ca^2+^ binding induces a conformational change in sorcin that promotes translocation from a soluble into a membrane-associated state. Its low abundance at cold-T_b_ can be explained by a general transition to a membrane-associated state where it would not be collected in the soluble fraction of the homogenate analyzed here. In rat caudate-putamen nucleus (CPN) sorcin is localized with smooth endoplasmic reticulum (SER) and mitochondria, two Ca^2+^-storing organelles [37]. In heart, sorcin interacts with ryanodine (RyR2) receptors to inhibit Ca^2+^ release, thereby reducing the gain of excitation-contraction coupling [38], [39]. Sorcin does co-localize with RyR3 in rat CPN [37] although whether it plays the identical role in Ca^2+^ homeostasis as it does with heart is not known. Control of Ca^2+^ release by sorcin at low temperature may also feature in the resistance of ground squirrel brain tissue [40] to in vitro aglycemic stress. Cell stress events such as low glucose and free radical exposure disrupt ER function, creating an impaired protein folding response and potentially leading to apoptosis. ER is also critical in mediating ischemic brain injury. Although the mechanism is not fully resolved, inhibition of ER stress has been suggested as neuroprotective following ischemia-reperfusion injury [41]. A disruption of Ca^2+^ homeostasis in ER is sufficient to induce apoptosis [42]. Suppression of Ca^2+^ release by pharmaceuticals such as dantrolene confers neuroprotection [42]. If sorcin’s low abundance at cold-T_b_ relates to a membrane-bound configuration, which limits Ca^2+^ release from ER during torpor and the high-stress rewarming period, such relocation of sorcin may protect against apoptosis and ER-mediated excitotoxicity.

### Cell Structure across the Hibernation Season

Cytoskeletal signals dominate this dataset. The cytoskeleton or its regulation was functionally enriched (Table 1) among proteins following the two most commonly observed abundance patterns (Fig. 2). Further, more than half of the proteins with ≥1.5 fold change and identified as important for separating the states by Random Forest were cytoskeletal components or binding proteins (Fig. 5A). Changes in neural cell architecture across torpor-arousal cycles are a well-documented aspect of hibernation biology [11], [13], [14]. Neural retraction occurs during every torpor bout [11] with re-establishment of neural profiles and function during each interbout euthermic period [11], [13], [14]. The ER likely plays a role in this process as well, as it controls local Ca^2+^ levels in growth cones and synaptic compartments [42]. Ca^2+^ is a key 2^nd^ messenger in growth cones, and decreased Ca^2+^ ER-outflow (via dantrolene) reduces axonal outgrowth [42]. The inverse, a relative increase in ER-Ca^2+^ release during euthermy would support the neural recovery observed after each torpor cycle and is consistent with the patterns noted for the Ca^2+^-regulating protein sorcin. This overrepresentation of cytoskeletal proteins that change seasonally and more importantly within torpor-arousal cycles supports the idea that synaptic remodeling is a significant neuroprotective feature in this portion of brain. Neuroprotection in this manner may derive from or promote the quiescence observed in this region during torpor and explain the absence of similarly abundant cytoskeletal proteins in a dataset from the more active brainstem [18]. mRNAs involved in cytoskeletal remodeling are also overrepresented in cerebral cortex, but not in the constitutively active hypothalamus [36]. Interestingly, no overlap exists between observed changes in protein (this study) and mRNA levels [36], however, many mRNAs corresponded to extracellular matrix proteins, a cell component excluded from our soluble protein preparation.

Despite the dominance of T_b_ or correlated physiological regulation observed in the forebrain proteome of 13-lined ground squirrels, we also uncovered limited examples of seasonal regulation. For instance, all detected isoforms of neurofilament heavy-chain (NEFH) displayed a seasonal winter increase (Fig. 5A). Neurofilaments are the most prominent cytoskeletal component of neurons in adults and occur as heteropolymers containing light- medium- and heavy-chain polypeptides. They are a major structural component of neurons, specifically implicated in maintaining their diameters and therefore nerve conduction velocities [43]. Neurofilament accumulation in non-hibernators is generally pathological and occurs in association with several conditions, including amyotrophic lateral sclerosis (ALS). We postulate that the winter-elevation of NEFH is a general signal of seasonal cytoskeletal plasticity and limited protein synthesis during euthermic periods to support neuronal remodeling.

Microtubules are another component of the neural cytoskeleton that are regulated across hibernation. Along with the actin cytoskeleton, controlled transport, development and extension of microtubule arrays are important for axonal growth [44], dendritic spine development and therefore synaptic plasticity [45], [46]. Microtubules are dynamic polymers of α- and β-tubulin dimers. Several isoforms of both α- and β-tubulins displayed overall summer-winter abundance shifts, but remained consistent throughout winter (e.g., TUBA1A^1461^, Fig. 5A). Two forms of β-tubulin (TUBB2A^1641^, TUBB4B^1527^) peaked in summer and were consistently winter-depressed. Only a single TUBA1C^1396^ isoform was identified, which was also consistently lowest in winter. However, several isoforms of α-tubulin-1A chain (TUBA1A) were identified (Table S2). These isoforms occurred in two reciprocal patterns, having intra-seasonal consistency, but an abundance change between the homeothermic and heterothermic periods. This observation suggests that the PTM status of this protein changes seasonally, with the natural dynamic instability of microtubules in homeothermy adjusted to handle the additional requirements of heterothermic plasticity. Our phosphoprotein staining experiment does indicate that several of the detected α-tubulin isoforms are phosphorylated, however this protein presents on 2D gels as several large spot trains and the location of phosphorylated isoforms within the trains suggests that this is not the sole regulatory PTM. Indeed, many PTM are known for tubulins [47], [48] and phosphorylation is not the most common form [48]. Our initial finding suggests that tubulins, and α-tubulin in particular may be an avenue for further study of winter neuroprotection.

### Torpor-arousal Cycle Regulation of Microtubules

The retraction of dendritic spines and synaptic structures at cold temperatures, such as during torpor is well documented in hibernators and non-hibernators alike [49], [50] and is likely driven by cold-induced disassembly of microtubules [12]. Dendritic spines are capable of regeneration upon re-warming in non-hibernators [49] but this process occurs more rapidly in hibernators [11]. In fact, the rate of dendrite extension during arousal from torpor occurs more quickly than rates observed in development or in response to environmental enrichment in non-hibernators [11]. If cytoskeletal components themselves display within-winter consistency, neuroprotection may instead exist in the form of regulatory proteins that are activated at specific points across heterothermy.

One contender is the cytoskeletal assembly regulator stathmin 1 (STMN1). Two stathmin isoforms (#2893, #2896) were the two most significantly changing proteins across the hibernation cycle (Table S4) and among the most important predictors of group classification by Random Forests. The abundance of both stathmin isoforms is high in cold-T_b_ states and consistently low otherwise (Fig. 5A). Microtubule polymerization is subject to dynamic instability, or rapid cycling between growth (‘rescue’) and disassembly (‘catastrophe’). The overall assembly or disassembly of microtubule arrays results from an imbalance in dynamic instability favoring either catastrophe or rescue. Polymerization is in part determined by the cytoplasmic availability of free tubulin [51]. Stathmin binds two tubulin dimers and thus removes them from the free soluble pool [52], [53], promoting microtubule disassembly by increasing the frequency of catastrophe. Additionally, stathmin can bind directly to microtubules [54], [55], acting primarily on the minus end but capable of increasing the frequency of catastrophe at both microtubule ends [55]. Stathmin’s tubulin-binding ability is regulated by PTM, specifically it is inhibited by phosphorylation at up to four serine sites [56]. Efforts to unequivocally determine the phosphorylation status of the stathmin isoforms identified here were unsuccessful. Neither 1D nor 2D western immunoblotting was sufficient to quantify total stathmin. The isoforms appeared dephosphorylated when evaluated by phosphoprotein stain and by pSer antibody labeling in 1D or 2D western blots, however this result is difficult to validate in the absence of definitive stathmin antibody labeling, likely an effect of the very low abundance of this protein in our forebrain sample. Thus the regulatory role of stathmin remains unclear. However, if dephosphorylated stathmin is most abundant at cold-T_b_, its absence due to proteolysis or inhibition by phosphorylation during euthermic periods would aid microtubule assembly.

Neural retraction/regeneration via microtubule arrays is also regulated by dihydropyrimidinase-related protein 2 (DPYSL2, synonymous with collapsin response mediator protein 2, or CRMP-2). DPYSL2 was important in group classification by Random Forests and differed significantly across hibernation states. This protein is highly expressed in the developing nervous system, and strongly downregulated in the adult brain with observed expression only in areas that retain plasticity [57]. The retention of a developmental phenotype in the forebrain of adult hibernating 13-lined ground squirrels is a feature which has previously been documented in the gut [58] and consistent with the retention of neural plasticity. DPYSL2 is crucial for axon/dendrite fate determination and is ultimately enriched in the distal portion of elongating/branching axons and during synaptogenesis [59], suggesting a role for DPYSL2 in the re-establishment of pre-synaptic termini. DPYSL2 promotes microtubule assembly by binding and delivering tubulin heterodimers to the plus end of growing microtubules, then co-polymerizes with tubulin [60]. This protein presents as multiple isoforms on 2D gels, supporting two abundance patterns (Fig. 6). Spot abundance is related to largely to T_b_, with certain isoforms more prevalent in either LT/EAr or consistently across the euthermic states (Fig. 6A). A series of phosphorylations at multiple sites inhibit DPYSL2 by impacting its ability to bind tubulin [60] and protein spots sharing the same abundance pattern also share a phosphorylation state (Fig. 6A–C). The two observed reciprocal patterns therefore likely represent relatively active and inactive states of the protein. The inactive, phosphorylated form is highest in LT/EAr, while the more active form is predominant during euthermy (Fig. 6D).

### Conclusions

Our results provide mechanistic insight into three aspects of neuroprotection in the forebrain of 13-lined ground squirrels; metabolic rate, cytoskeletal dynamics and Ca^2+^ regulation. First, although it is acknowledged that metabolic rate depression is necessary to balance the ∼90% reduction in cerebral perfusion during torpor, we documented that few changes occur among metabolic enzymes in this brain region between summer and winter, or across winter heterothermy. We therefore postulate that the ground squirrel forebrain exploits passive temperature reduction via the Q_10_ effect to depress metabolic rates and cell functions during torpor. Subsequent elements of neuroprotection are likely necessary to both stabilize the cell at low T_b_, and to shepherd damage-free recovery from the torpid state.

Second, our data provide insight into the molecular mechanisms that both stabilize cells in the cold and protect them during arousal. The majority of proteins that vary with hibernation physiology were associated with the cytoskeleton, highlighting its importance in defining the torpid and aroused states. While cytoskeletal proteins themselves appear to differ from summer across the entire heterothermic period, their regulatory factors track changes in T_b_. Both cytoskeletal regulators highlighted here are affected by PTM [56], [60], specifically phosphorylation; we have demonstrated a phosphorylation shift associated with isoforms of opposing abundance patterns in one of these, DPYSL2. It is not surprising that phospho-modifications could be temperature-dependent regulatory mechanisms, as this process is rapid, reversible and unaffected by the dramatically decreased transcription and translation in place at low T_b_ [27], [28]. Indeed, phosphorylation also regulates the function of tau, another microtubule binding protein implicated in cytoskeletal remodeling during hibernation [61], [62]. It is noteworthy that both regulatory proteins discussed here (STMN1 & DPYSL2) differ from other microtubule associated proteins including tau in that they more efficiently bind tubulin heterodimers as opposed to microtubules.

Third, these data also highlight Ca^2+^ homeostasis as an element of cell stabilization during torpor-arousal cycles. Ca^2+^ signaling has wide-reaching effects in regulating general cell functions. At least one protein implicated in Ca^2+^ homeostasis (SRI, sorcin) differed in the soluble fraction of 13-lined ground squirrel forebrain in response to hibernation, and specifically T_b_. This reveals an element of the strategy in place to regulate intracellular Ca^2+^ handling during torpor.

It is clear that despite relatively few proteomic differences in forebrain across the hibernator’s annual cycle, key proteins do change. Body temperature is the most relevant factor in defining protein abundance changes, as evidenced by the temperature segregation of the mixed-physiology Fall animals. This suggests that the forebrain manages homeostasis during its quiescent torpor periods distinctly from interbout arousals. Components of neuroprotection in hibernation revealed by their response to T_b_ may be useful therapeutic targets for human neuropathies.

## Supporting Information

Table S1 Biometrics and housing information for Fall Transition 13-lined ground squirrels used in this study.
(XLS)

Table S2 Summary of all unequivocally identified proteins that differed among six base physiological states of hibernation in 13-lined ground squirrels.
(XLSX)

Table S3 Summary of all unequivocally identified proteins that differed among six base physiological states of hibernation and the Fall Transition in 13-lined ground squirrels.
(XLSX)

Table S4 All protein identity results. Data are presented for supporting amino acid sequences from homologous proteins for protein spots determined to be significant by ANOVA after FDR or important input spots from Random Forests clustering. Multiple potential protein identities are also listed when appropriate.
(XLSX)

Table S5 Statistical summary for all unequivocally identified proteins (n = 56), which differed significantly among defined physiological states after multiple test correction.
(XLSX)

Table S6 Statistical summary for all unequivocally identified proteins (n = 54), which differed among the defined physiological states and the Fall Transition animals.
(XLSX)

Table S7 Data summary for all unequivocally identified proteins (n = 62), which differed significantly for either one-way analysis after multiple ANOVA test correction.
(XLSX)

Table S8 Normalized protein spot abundance data from all sampled 13-lined ground squirrels. Group identifies sampled state abbreviation and ID reflects individual animal identifier. Remaining column headings denote spot number on the master gel and official gene symbol. Data are presented for all identified spots, selected by significance in any statistical procedure or in select cases as non-significant but robust control spots.
(XLSX)

## References

[1] Pengelley ET, Fisher KC (1961) Rhythmical arousal from hibernation in the golden-mantled ground squirrel, *Citellus lateralis tescorum* . Can J Zool 39: 105–120.

[2] Andrews MT (2007) Advances in molecular biology of hibernation in mammals. Bioessays 29: 431–440. 17450592 10.1002/bies.20560

[3] Carey HV, Andrews MT, Martin SL (2003) Mammalian hibernation: cellular and molecular responses to depressed metabolism and low temperature. Physiol Rev 83: 1153–1181. 14506303 10.1152/physrev.00008.2003

[4] Lyman CP, Willis JS, Malan A, Wang LCH (1982) Hibernation and Torpor in Mammals and Birds; Kozlowski TT, editor. New York: Academic Press. 317 p.

[5] Dave KR, Christian SL, Perez-Pinzon MA, Drew KL (2012) Neuroprotection: lessons from hibernators. Comp Biochem Physiol B 162: 1–9. 22326449 10.1016/j.cbpb.2012.01.008 PMC3334476

[6] Drew KL, Buck CL, Barnes BM, Christian SL, Rasley BT, et al (2007) Central nervous system regulation of mammalian hibernation: implications for metabolic suppression and ischemia tolerance. J Neurochem 102: 1713–1726. 17555547 10.1111/j.1471-4159.2007.04675.x PMC3600610

[7] Frerichs KU, Kennedy C, Solokoff L, Hallenbeck JM (1994) Local cerebral blood flow during hibernation, a model of natural tolerance to "cerebral ischemia". J Cereb Blood Flow Metab 14: 193–205. 8113316 10.1038/jcbfm.1994.26

[8] Drew KL, Toien O, Rivera PM, Smith MA, Perry G, et al (2002) Role of the antioxidant ascorbate in hibernation and warming from hibernation. Comp Biochem Physiol C Toxicol Pharmacol 133: 483–492. 12458177 10.1016/s1532-0456(02)00118-7

[9] Frerichs KU (1999) Neuroprotective strategies in nature-novel clues for the treatment of stroke and trauma. Acta Neurochir Suppl 73: 57–61. 10.1007/978-3-7091-6391-7_9 10494342

[10] van Breukelen F, Martin SL (2002) Molecular adaptations in mammalian hibernators: unique adaptations or generalized responses? J Appl Physiol 92: 2640–2647. 12015384 10.1152/japplphysiol.01007.2001

[11] von der Ohe CG, Darian-Smith C, Garner CC, Heller HC (2006) Ubiquitous and temperature-dependent neural plasticity in hibernators. J Neurosci 26: 10590–10598. 17035545 10.1523/JNEUROSCI.2874-06.2006 PMC6674705

[12] Kirschner MW, Williams RC, Weingarten M, Gerhart JC (1974) Microtubules from mammalian brain: some properties of their depolymerization products and a proposed mechanism of assembly and disassembly. Proc Natl Acad Sci U S A 71: 1159–1163. 4524627 10.1073/pnas.71.4.1159 PMC388183

[13] Popov VI, Bocharova LS, Bragin AG (1992) Repeated changes of dendritic morphology in the hippocampus of ground squirrels in the course of hibernation. Neuroscience 48: 45–51. 1584424 10.1016/0306-4522(92)90336-z

[14] Popov VI, Bocharova LS (1992) Hibernation-induced structural changes in synaptic contacts between mossy fibres and hippocampal pyramidal neurons. Neuroscience 48: 53–62. 1584425 10.1016/0306-4522(92)90337-2

[15] von der Ohe CG, Garner CC, Darian-Smith C, Heller HC (2007) Synaptic protein dynamics in hibernation. J Neurosci 27: 84–92. 17202475 10.1523/JNEUROSCI.4385-06.2007 PMC6672296

[16] Russell RL, O'Neill PH, Epperson LE, Martin SL (2010) Extensive use of torpor in 13-lined ground squirrels in the fall prior to cold exposure. J Comp Physiol B 180: 1165–1172. 20556614 10.1007/s00360-010-0484-8 PMC3116921

[17] Hindle AG, Karimpour-Fard A, Epperson LE, Hunter LE, Martin SL (2011) Skeletal muscle proteomics: carbohydrate metabolism oscillates with seasonal and torpor-arousal physiology of hibernation. Am J Physiol Regul Integr Comp Physiol 301: R1440–R1452. 21865542 10.1152/ajpregu.00298.2011 PMC3213940

[18] Epperson L, Rose J, Russell R, Nikrad M, Carey H, et al (2010) Seasonal protein changes support rapid energy production in hibernator brainstem. J Comp Physiol B 180: 599–617. 19967378 10.1007/s00360-009-0422-9 PMC3116658

[19] Benjamini Y, Hochberg Y (1995) Controlling the false discovery rate: a practical and powerful approach to multiple testing. J R Stat Soc Series B Stat Methodol 57: 289–300.

[20] Breiman L (2001) Random Forests. Mach Learn 45: 5–32.

[21] Liaw A, Wiener M (2002) Classification and regression by randomForest. R News 2: 18–22.

[22] Diaz-Uriarte R (2010) varSelRF: variable selection using random forests. R package version 0.7–3. http://CRAN.R-project.org/package=varSelRF.

[23] R Development Core Team (2012) R: a language and environment for statistical computing. R Foundation for Statistical Computing, Vienna, Austria. ISBN 3–900051–07–0, URL http://www.R-project.org/.

[24] Yan J, Barnes BM, Kohl F, Marr TG (2008) Modulation of gene expression in hibernating arctic ground squirrels. Physiol Genomics 32: 170–181. 17925484 10.1152/physiolgenomics.00075.2007

[25] Huang DW, Sherman BT, Lempicki RA (2009) Systematic and integrative analysis of large gene lists using DAVID bioinformatics resources. Nat Protoc 4: 44–57. 19131956 10.1038/nprot.2008.211

[26] Agrawal GK, Thelen JJ (2005) Development of a simplified, economical polyacrylamide gel staining protocol for phosphoproteins. Proteomics 5: 4684–4688. 16267815 10.1002/pmic.200500021

[27] van Breukelen F, Martin SL (2001) Translational initiation is uncoupled from elongation at 18C during mammalian hibernation. Am J Physiol Regul Integr Comp Physiol 281: R1374–1379. 11641105 10.1152/ajpregu.2001.281.5.R1374

[28] van Breukelen F, Martin SL (2002) Reversible depression of transcription during hibernation. J Comp Physiol B 172: 355–361. 12122451 10.1007/s00360-002-0256-1

[29] Grabek KR, Karimpour-Fard A, Epperson LE, Hindle AG, Hunter LE, et al (2011) Multistate proteomics analysis reveals novel strategies used by a hibernator to precondition the heart and conserve ATP for winter heterothermy. Physiol Genomics 43: 1263–1275. 21914784 10.1152/physiolgenomics.00125.2011 PMC3217319

[30] Jani A, Epperson E, Martin J, Pacic A, Ljubanovic D, et al (2011) Renal protection from prolonged cold ischemia and warm reperfusion in hibernating squirrels. Transplantation 92: 1215–1221. 22082817 10.1097/TP.0b013e3182366401

[31] Samland AK, Sprenger GA (2009) Transaldolase: from biochemistry to human disease. The Int J Biochem Cell Biol 41: 1482–1494. 19401148 10.1016/j.biocel.2009.02.001

[32] Drew KL, Osborne PG, Frerichs KU, Hu Y, Koren RE, et al (1999) Ascorbate and glutathione regulation in hibernating ground squirrels. Brain Res 851: 1–8. 10642822 10.1016/s0006-8993(99)01969-1

[33] Epperson LE, Rose JC, Carey HV, Martin SL (2010) Seasonal proteomic changes reveal molecular adaptations to preserve and replenish liver proteins during ground squirrel hibernation. Am J Physiol Regul Integr Comp Physiol 298: R329–R340. 19923364 10.1152/ajpregu.00416.2009 PMC2828287

[34] Geiser F (2004) Metabolic rate and body temperature reduction during hibernation and daily torpor. Ann Rev Physiol 66: 239–274. 14977403 10.1146/annurev.physiol.66.032102.115105

[35] Andrews MT, Russeth KP, Drewes LR, Henry PG (2009) Adaptive mechanisms regulate preferred utilization of ketones in the heart and brain of a hibernating mammal during arousal from torpor. Am J Physiol Regul Integr Comp Physiol 296: R383–R393. 19052316 10.1152/ajpregu.90795.2008 PMC2643978

[36] Schwartz C, Hampton M, Andrews MT (2013) Seasonal and regional differences in gene expression in the brain of a hibernating mammal. PLoS ONE 8: e58427. 23526982 10.1371/journal.pone.0058427 PMC3603966

[37] Pickel VM, Clarke CL, Meyers MB (1997) Ultrastructural localization of sorcin, a 22 kDa calcium binding protein, in the rat caudate-putamen nucleus: association with ryanodine receptors and intracellular calcium release. J Comp Neurol 386: 625–634. 9378856 10.1002/(sici)1096-9861(19971006)386:4<625::aid-cne8>3.0.co;2-4

[38] Lokuta AJ, Meyers MB, Sander PR, Fishman GI, Valdivia HH (1997) Modulation of cardiac ryanodine receptors by sorcin. J Biol Chem 272: 25333–25338. 9312152 10.1074/jbc.272.40.25333

[39] Farrell EF, Antaramian A, Rueda A, Gómez AM, Valdivia HH (2003) Sorcin Inhibits calcium release and modulates excitation-contraction coupling in the heart. J Biol Chem 278: 34660–34666. 12824171 10.1074/jbc.M305931200

[40] Frerichs KU, Hallenbeck JM (1998) Hibernation in ground squirrels induces state and species-specific tolerance to hypoxia and aglycemia: an in vitro study in hippocampal slices. J Cereb Blood Flow Metab 18: 168–175. 9469159 10.1097/00004647-199802000-00007

[41] Nakka VP, Gusain A, Raghubir R (2010) Endoplasmic reticulum stress plays critical role in brain damage after cerebral ischemia/reperfusion in rats. Neurotox Res 17: 189–202. 19763736 10.1007/s12640-009-9110-5

[42] Mattson MP, LaFerla FM, Chan SL, Leissring MA, Shepel PN, et al (2000) Calcium signaling in the ER: its role in neuronal plasticity and neurodegenerative disorders. Trends Neurosci 23: 222–229. 10782128 10.1016/s0166-2236(00)01548-4

[43] Yuan A, Rao MV, Veeranna, Nixon RA (2012) Neurofilaments at a glance. J Cell Sci 125: 3257–3263. 22956720 10.1242/jcs.104729 PMC3516374

[44] Baas PW (1997) Microtubules and axonal growth. Current Opinion in Cell Biology 9: 29–36. 9013665 10.1016/s0955-0674(97)80148-2

[45] Jaworski J, Kapitein LC, Gouveia SM, Dortland BR, Wulf PS, et al (2009) Dynamic microtubules regulate dendritic spine morphology and synaptic plasticity. Neuron 61: 85–100. 19146815 10.1016/j.neuron.2008.11.013

[46] Gu J, Firestein BL, Zheng JQ (2008) Microtubules in dendritic spine development. J Neurosci 28: 12120–12124. 19005076 10.1523/JNEUROSCI.2509-08.2008 PMC2605155

[47] Janke C, Bulinski JC (2011) Post-translational regulation of the microtubule cytoskeleton: mechanisms and functions. Nat Rev Mol Cell Biol 12: 773–786. 22086369 10.1038/nrm3227

[48] Wloga D, Gaertig J (2010) Post-translational modifications of microtubules. J Cell Sci 123: 3447–3455. 20930140 10.1242/jcs.063727 PMC2951466

[49] Kirov SA, Petrak LJ, Fiala JC, Harris KM (2004) Dendritic spines disappear with chilling but proliferate excessively upon rewarming of mature hippocampus. Neuroscience 127: 69–80. 15219670 10.1016/j.neuroscience.2004.04.053

[50] Roelandse M, Matus A (2004) Hypothermia-associated loss of dendritic spines. J Neurosci 24: 7843–7847. 15356196 10.1523/JNEUROSCI.2872-04.2004 PMC6729919

[51] Gardner MK, Zanic M, Howard J (2013) Microtubule catastrophe and rescue. Curr Opin Cell Biol 25: 14–22. 23092753 10.1016/j.ceb.2012.09.006 PMC3556214

[52] Belmont LD, Mitchison TJ (1996) Identification of a protein that interacts with tubulin dimers and increases the catastrophe rate of microtubules. Cell 84: 623–631. 8598048 10.1016/s0092-8674(00)81037-5

[53] Jourdain L, Curmi P, Sobel A, Pantaloni D, Carlier M-F (1997) Stathmin: a tubulin-sequestering protein which forms a ternary T_2_S complex with two tubulin molecules. Biochemistry 36: 10817–10821. 9312271 10.1021/bi971491b

[54] Howell B, Larsson N, Gullberg M, Cassimeris L (1999) Dissociation of the tubulin-sequestering and microtubule catastrophe-promoting activities of oncoprotein 18/stathmin. Mol Biol Cell 10: 105–118. 9880330 10.1091/mbc.10.1.105 PMC25157

[55] Manna T, Thrower D, Miller HP, Curmi P, Wilson L (2006) Stathmin strongly increases the minus end catastrophe frequency and induces rapid treadmilling of bovine brain microtubules at steady state in vitro. J Biol Chem 281: 2071–2078. 16317007 10.1074/jbc.M510661200

[56] Manna T, Thrower DA, Honnappa S, Steinmetz MO, Wilson L (2009) Regulation of microtubule dynamic instability in vitro by differentially phosphorylated stathmin. J Biol Chem 284: 15640–15649. 19359244 10.1074/jbc.M900343200 PMC2708860

[57] Charrier E, Reibel S, Rogemond V, Aguera M, Thomasset N, et al (2003) Collapsin response mediator proteins (CRMPs). Mol Neurobiol 28: 51–63. 14514985 10.1385/MN:28:1:51

[58] Martin SL, Epperson LE, Rose JC, Kurtz CC, Ane C, et al (2008) Proteomic analysis of the winter-protected phenotype of hibernating ground squirrel intestine. Am J Physiol Regul Integr Comp Physiol 295: R316–328. 18434441 10.1152/ajpregu.00418.2007

[59] Schmidt EF, Strittmatter SM (2007) The CRMP family of proteins and their role in Sema3A signaling. In: Pasterkamp RJ, editor. Semaphorins: Receptor and Intracellular Signaling Mechanisms: Springer New York. 1–11. 10.1007/978-0-387-70956-7_1 PMC2853248 17607942

[60] Khanna R, Wilson SM, Brittain JM, Weimer J, Sultana R, et al (2012) Opening Pandora's jar: a primer on the putative roles of CRMP2 in a panoply of neurodegenerative, sensory and motor neuron, and central disorders. Future Neurol 7: 749–771. 23308041 10.2217/FNL.12.68 PMC3539824

[61] Arendt T, Stieler J, Strijkstra AM, Hut RA, Rudiger J, et al (2003) Reversible paired helical filament-like phosphorylation of tau is an adaptive process associated with neuronal plasticity in hibernating animals. J Neurosci 23: 6972–6981. 12904458 10.1523/JNEUROSCI.23-18-06972.2003 PMC6740664

[62] Stieler JT, Bullmann T, Kohl F, Tøien Ø, Brückner MK, et al (2011) The physiological link between metabolic rate depression and tau phosphorylation in mammalian hibernation. PLoS ONE 6: e14530. 21267079 10.1371/journal.pone.0014530 PMC3022585

